# Being mindful in the tax context in Italy: Examining whether and how mindfulness relates with tax evasion intentions and support for tax progressivity

**DOI:** 10.1371/journal.pone.0253627

**Published:** 2021-06-25

**Authors:** Valeria De Cristofaro, Mauro Giacomantonio, Valerio Pellegrini, Marco Salvati, Luigi Leone

**Affiliations:** Department of Social and Developmental Psychology, Sapienza University of Rome, Rome, Italy; Universidad Loyola Andalucia Cordoba, SPAIN

## Abstract

Two studies explored whether and how mindfulness relates with citizens’ tax evasion intentions and support for progressive tax rates. Based on theoretical and empirical grounds, in Study 1 (*N* = 1,175) we proposed that mindfulness would be negatively related with tax evasion intentions through decreased social dominance orientation. Drawing on Duckitt’s dual-process motivational model, in Study 2 (*N* = 722) we proposed that mindfulness would be positively related with support for progressive taxation through the mediation of lower competitive-jungle beliefs, and then lower social dominance orientation. Instead, we did not expect to find mediation of the link between mindfulness and support for progressive taxation through dangerous-world beliefs and right-wing authoritarianism. These studies inform about the motivational pathways through which mindfulness relates with tax evasion intentions and support for progressive taxation.

## Introduction

Economic inequality is one of the major societal issues of our time. The gap between the world’s poorest and wealthiest has widened in recent decades, with the Covid-19 pandemic unmasking and amplifying economic inequality in society as well as between nations [1].

The negative effects of economic inequality have been widely documented. There is evidence, for example, that highly unequal societies are characterized by high levels of infant mortality, limited access to education, poor health care provision, and reduced life expectancy [2]. Research has demonstrated that economic inequality has negative consequences for people’s health and well-being [3–5]. Also, it has been shown that economic inequality reduces trust and social capital, whereas it increases violence and social unrest [6].

The present research aims at examining the psychological factors and processes that help to maintain versus reduce economic inequality. Specifically, this research aims at examining whether and how mindfulness, or moment-by-moment awareness, relates with citizens’ (a) tax evasion intentions (as an instance of inequality-enhancing behavior) and (b) support for progressive taxation (as an instance of inequality-reducing behavior). Starting from the assumption and empirical evidence that mindfulness promotes prosocial behavior such as empathy, helping behavior, and ethical decision making [7,8], we investigated whether and how it relates with citizens’ responsiveness to tax laws. This is relevant for both policies and research as it is a key issue in the study of economic inequality, both in terms of socioeconomic development and for public welfare and well-being [9,10].

The paper is organized as follows: The next section presents the construct of mindfulness and the theoretical framework for studying its role in the context of taxation and, more generally, economic inequality. This is followed by discussion of the motivational paths through which mindfulness may be linked to tax evasion intentions and support for tax progressivity, divided into Studies 1 and 2. The final sections summarize and conclude the paper.

## Mindfulness

According to Brown and Ryan (2003) [12], mindfulness refers to people’s tendency to self-regulate their attention and to be aware of internal and external stimuli, accepting them in a nonjudgmental way. Mindfulness can be reached via specific training aimed at improving people’s ability to “stay in touch” with whatever they are experiencing moment by moment [11]. Alternatively, and as in the present research, mindfulness can be considered as a dispositional mental trait that can be measured by specific self-report scales [12,13].

Ongoing research increasingly supports the notion that individual differences in trait mindfulness positively relate with prosocial [14], pro-environmental [15], and pro-organizational [16,17] behaviors. People high in trait mindfulness were found to be more empathic and concerned with others’ well-being [7] and more willing to act ethically, to value upholding ethical standards, and to make ethical decisions [8]. Helm and Subramaiam (2019) found that mindfulness favors sustainable consumption behavior through decreased adoption of materialistic values [18,19]. There is also evidence that mindfulness promotes helping behavior [7,20] and prevents ostracism [21]. In addition, and of present relevance, Panno and colleagues (2018) reported that mindfulness leads people to behave pro-environmentally by reducing their adherence to social dominance orientation (SDO), the people’s tendency to support hierarchical intergroup relationships [22,23]. These authors, employing undergraduate students and meditation practitioners versus nonpractitioners, found that mindful people were less likely to approve hierarchical societal relationships (i.e., SDO) and then, they were more willing to engage in pro-environmental attitudes and behaviors [22].

In the present study, we relied on the aforementioned research on mindfulness and proposed that mindfulness may be related to citizens’ attitudes and behavioral tendencies toward taxes and the tax system. Specifically, in line with the findings of Panno et al. (2018) [22], we examined whether (a) being mindful may relate with citizens’ tax evasion intentions, (b) mindfulness relates to support for a progressive tax system, and (c) these relations are mediated through SDO.

## Mindfulness and tax evasion

Tax evasion is one of the major public policy problems, with negative consequences on compliant taxpayers, infrastructure spending, health care and services, and the entire safety net. Revenue losses from evasion are particularly critical for the maintenance of adequate social services and sustainable economies [24,25]. Tax evasion distorts the principle of perfect market resource allocation [26]; it weakens the cardinal virtue of social justice and, consequently, poses a fundamental threat to the reduction of economic inequality [27,28]. Importantly, past research on tax compliance has found that tax evasion is associated with a pro-self (vs. pro-social) orientation. Because of their tendency to maximize one’s individual gains, pro-self (vs. pro-social) oriented citizens are more likely to evade taxes, thereby supporting economic inequality in favor of their privileged position [29].

Based on existing literature on pro-social effects of mindfulness [12], the first goal of the present research is to investigate whether and how mindfulness relates with citizens’ intentions to pay or evade taxes. More specifically, following Panno and colleagues (2018) [22], we proposed that the relation between mindfulness and tax evasion may be explained by SDO. According to these authors, indeed, mindful people are less inclined to adhere to SDO due to their tendency to act ethically and to display empathy and other-oriented behaviors. Consequently, lower SDO is negatively associated with inequality-related outcomes [30–32]. This is because SDO reflects a preference for antagonism and the supremacy of powerful societal members over weaker ones; people with an elevated SDO endorse a hierarchical relationship between groups and a stratified vision of society [33,34]. From this starting point, we predicted that *mindfulness should negatively relate with citizens’ adherence to SDO and thus with their tax evasion intentions* [*Hypothesis 1* (*H1*)]. We tested this prediction in Study 1.

## Mindfulness and tax progressivity

To redress economic inequality, several political decisions must be made regarding how economic resources should be extracted and allocated, such as implementing progressive taxation [35]. Progressive taxation (i.e., the degree to which the tax rate is higher for the high-income earners than for the low-income earners) is a policy tool for changing the taxation system so that income and wealth are redistributed to the less fortunate [36] and economic inequality is reduced [37]. As such, support for a progressive tax system is associated with a pro-social (vs. pro-self) orientation. Indeed, pro-social (vs. pro-self) oriented individuals are concerned with others’ gains and losses and they tend to maximize joint and equal outcomes [29,38].

The second goal of the present research is to investigate whether and how mindfulness relates with citizens’ decision on whether to support or reject tax progressivity. More specifically, building on the role of SDO, we wanted to elaborate further on the motivational paths for how mindfulness may relate to citizens’ attitudes toward the tax system. To more solidly ground mindfulness in research on taxation and economic inequality, it is important to study these paths.

Accordingly, Study 2 built on the dual-process motivational model (DPM; [39]), which posits that inequality-related outcomes are linked with two distinct ideological attitudes which have different motivational bases: SDO and right-wing authoritarianism. As previously explained, SDO captures preference for hierarchical intergroup relationships [23]. In accordance with Duckitt et al. (2002) [39], SDO depends upon a competitive-jungle social worldview, a relatively stable belief that the social world is a competitive jungle in which the advantaged win and the disadvantaged lose [40,41]. Instead, right-wing authoritarianism (RWA) reflects a preference for the maintenance of in-group norms and values as well as existing traditions and conventions [42]. People espousing elevated right-wing authoritarianism support coercive social control, obedience, and respect for authorities [42]. As a consequence, RWA is related with hierarchy and inequality, but from an intragroup perspective, rather than an intergroup perspective (as is the case for SDO). In Duckitt’s model, RWA stems from a dangerous-world social worldview, a relatively stable belief that the social world is a dangerous and unpredictable place in which norms and values are perceived to be under threat [40,41], and—by the same token—norms, values, and personal honesty are perceived as the pillars of a cherished status quo [43]. Unlike SDO, then, RWA does not align strongly with ruthlessness in competing for resources and privilege.

Hence, in the present research, we assume that SDO positively relates to people’s tendency to compete with other societal members, driven by a stratified vision of society in which the powerful dominate over the weaker. Also, because of its focus on hierarchical intergroup relationships, SDO should be more likely to relate with mindfulness in comparison to RWA, which focuses on the maintenance of norms and values. Because of its focus on intergroup hierarchical relationships in the competition for status and resources, we proposed here that the relation between mindfulness and tax-related criteria would more decisively follow the competitive jungle–social dominance path, compared with the dangerous world–RWA path. Stated formally, *mindfulness should negatively relate with citizens’ competitive-jungle beliefs and then their SDO*. *In turn*, *lower SDO should positively relate with their support for tax progressivity* [*Hypothesis 2* (*H2*)]. People’s support for tax progressivity should be more strongly explained by this motivational path of competitive jungle–social dominance compared to the motivational path of RWA, which is based on dangerous-world beliefs. Put differently, the dangerous world–RWA path should be less central in explaining motivation to support tax progressivity in respect to the competitive jungle–social dominance path [*Hypothesis 2a* (*H2a*)]. In the motivational path of competitive jungle–social dominance, indeed, emphasis is put on power asymmetry and competition between individuals, which should strongly refrain individuals from supporting income and wealth redistribution through progressive taxation. Conversely, in the motivational path of dangerous world–authoritarianism, emphasis in put on personal values related to honesty as well as on unchanging tradition, which should be less associated with individuals’ decision to support or reject tax progressivity. Therefore, we expect that individuals’ support for progressive taxation should be mainly attributable to the motivational path of SDO and, to a lesser extent, to that of RWA.

Taken together, Studies 1 and 2 extend the literature on the pro-social effects of mindfulness [12] and deepen our knowledge about motivational paths underlying tax evasion intentions (as an instance of inequality-enhancing behavior) and support for progressive taxation (as an instance of inequality-reducing behavior). We describe each study in detail below.

## Study 1

Study 1 aimed to investigate the association between mindfulness and tax evasion intentions through SDO. Based on theory and evidence reviewed above [22], we expected that *mindfulness would be negatively related with SDO and*, *consequently*, *would be linked with lower tax evasion intentions* [*Hypothesis 1* (*H1*)].

### Participants

A total of 1,175 people living in Italy were contacted online and participated in the study on a voluntary basis. The sample consisted of 402 men (34.2%) and 773 women (65.8%), aged 14–89 years (*M* = 27.39, *SD* = 13.17). The educational level of participants varied from secondary school to PhD as follows: 10.3% secondary school, 66.5% high school, 4.2% bachelor’s degree, 18% master’s degree, and 1.0% PhD. Regarding political orientation, 29.4% of the sample classified themselves as left wing, 32.3% as center left, 21.1% as center, 14.8% as center right, and 8.3% as right wing. Anonymity was assured and written informed consent was obtained. Ethical approval was received by the ethics committee of the Department of Social and Developmental Psychology, Sapienza University of Rome.

### Measures and procedure

#### Mindfulness

Participants first completed the 15-item *Mindful Attention Awareness Scale* (*MAAS*; [12]), which assesses individual differences in receptive attention to and awareness of ongoing internal and external experiences. Examples of items are “I find it difficult to stay focused on what’s happening in the present” [reverse coded] and “I do jobs or tasks automatically, without being aware of what I’m doing” [reverse coded]. Ratings were made on 6-point scales (anchors: 1 = *almost never*; 6 = *almost always*). A composite mindfulness score was computed by averaging responses across items (*α* = .84); high values indicate people to be intrinsically high in mindfulness.

#### Social dominance orientation

Then, we asked participants to complete the 16-item *SDO scale* [23], designed to tap individual differences in SDO. Examples of items are: “Some groups of people are simply inferior to other groups” and “Inferior groups should stay in their place.” Ratings were made on 7-point scales, where 1 = *completely disagree* and 7 = *completely agree*. A composite SDO score was computed by averaging responses (*α* = .78); high values indicate higher SDO.

#### Tax evasion intentions

Next, participants completed the *Tax Evasion Subscale* (*TE*) of the *Tax Compliance Inventory* (*TAX-I*; [44]). This subscale consists of eight items, designed to measure intentions to evade taxes. Examples of items are: “You could declare your car as a company car, although your use of it is only 30% for business purposes, and at least 50% business use is required for it to be assessed as a company car. How likely is it that you would declare your car as a company car?” and “You could enter private journeys as company journeys in your driver’s logbook. How likely is it that you would enter private journeys as company ones?”. Ratings were made on 7-point scales, ranging from 1 = *completely unlikely* to 7 = *completely likely*. A composite tax evasion intentions score was computed by averaging responses (*α* = .91); high values indicate higher intentions to evade taxes.

### Results

Correlations among variables are displayed in Table 1.

**Table 1:** Intercorrelations, means, and standard deviations for scores on Mindfulness (MAAS), Social Dominance Orientation (SDO), and Tax Evasion intentions (TE).

	*M*	*SD*	(1)	(2)	(3)
(1) MAAS	4.11	.79	-		
(2) SDO	2.96	.81	-.13***	-	
(3) TE	2.85	1.68	-.17***	.22***	-

*Note*. Study 1 (*N* = 1175)

*** *p* < .001.

**Fig 1:**
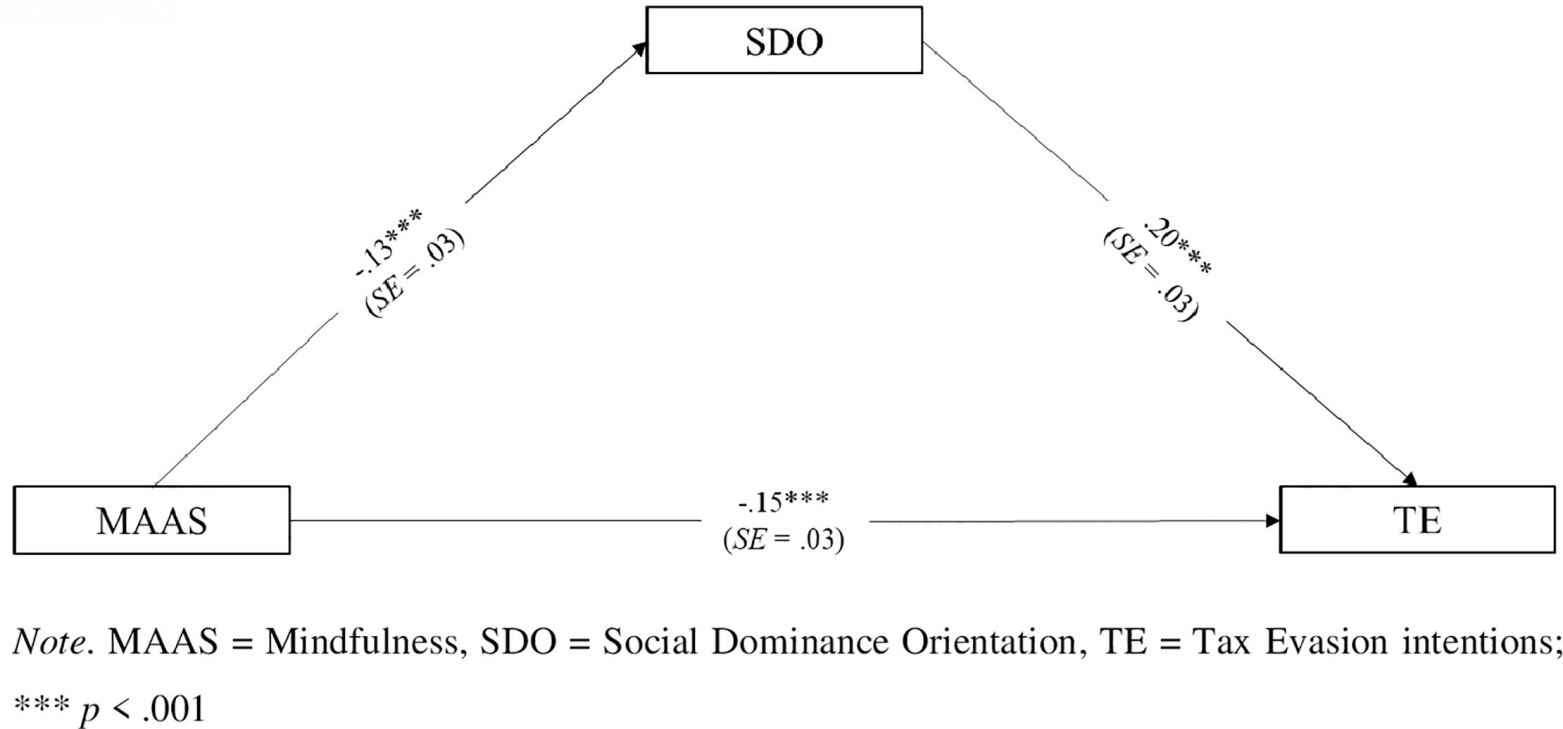
Mediation analysis model in Study 1 (*N* = 1,175): Standardized parameters.

We expected mindfulness to negatively relate with SDO and, consequently, with tax evasion intentions (*H1*). We tested our hypothesis by conducting a mediation analysis, using Hayes’s (2013) PROCESS macro (Model 4) with 5,000 bootstrap samples and 95% confidence intervals [45]. In the model, mindfulness was the independent variable (X), SDO was the mediator (M), and tax evasion intentions was the dependent variable (Y). The results of the mediation analysis are illustrated in Fig 1.

Mindfulness was negatively associated with SDO, *β* = -.13, *SE* = .03, *t* = -4.38, *p* < .001, *95% CI* [-.1837, -.0700], meaning that mindful people were less characterized by SDO. Also, SDO was positively associated with tax evasion intentions, *β* = .20, *SE* = .03, *t* = 7.20, *p* < .001, *95% CI* [.1487, .2600], meaning that people who possessed high levels of SDO were more inclined to evade taxes. Furthermore, mindfulness negatively related with tax evasion intentions, *β* = -.15, *SE* = .03, *t* = -5.22, *p* < .001, *95% CI* [-.2040, -.0926], indicating that as mindfulness increased, people were less inclined to evade taxes.

Importantly, the indirect relation between mindfulness and tax evasion intentions was negative and significant, *β* = -.02, *SE* = .007, *95% CI* [-.0422, -.0122]. Results of Study 1 showed that mindfulness related with tax evasion intentions directly as well as indirectly through lower levels of SDO. Although conventionally small, this indirect relation provided evidence for a partial mediation model [46], lending support for *H1*.

## Study 2

Study 2 aimed at extending Study 1 in two ways. First, we shifted our focus on the extent to which people supported (or rejected) a progressive system of taxation. Second, building on Duckitt’s (2002) [39] dual-process model, we investigated whether the positive relation between mindfulness and support for progressive taxation could be primarily attributable to the motivational path of competitive-jungle beliefs–SDO or, instead, to the motivational path of dangerous-world beliefs–RWA. Given mindfulness’s emphasis on pro-sociality [12], we see mindfulness as more closely aligned with the SDO path, which places emphasis on unrestrained pursuit of hierarchy-enhancing pro-self behaviors, compared to the RWA path, which places emphasis on intolerance for deviations, but also to rule abidance, restraint, and honesty [43]. More specifically, because mindfulness promotes cultivation of empathy and ethical decision making [20], we expected that *mindfulness would positively relate with support for a progressive taxation through lower competitive-jungle beliefs and then*, *SDO* [*Hypothesis 2* (*H2*)]. On the contrary, we expected a weaker pathway through dangerous-world beliefs and RWA [*Hypothesis 2a* (*H2a*)].

### Participants

Participants were contacted online and participated in the study on a voluntary basis. In total, we recruited 722 people living in Italy. Of these, 240 were men (33.2%) and 482 were women (66.8%), aged 14–73 years (*M* = 27.18, *SD* = 13.23). The educational level of participants varied as follows: 0.4% primary school, 7.8% secondary school, 76.2% high school, 6.6% bachelor’s degree, 5.5% master’s degree, and 3.5% PhD. Regarding political orientation, 28.5% of participants classified themselves as left wing, 30.2% as center left, 19.1% as center, 15.4% center right, and 6.8% right wing. Anonymity was assured and written informed consent was obtained. Ethical approval was received by the ethics committee of the Department of Social and Developmental Psychology, Sapienza University of Rome.

### Measures and procedure

#### Mindfulness

Participants first completed the 15-item *Mindful Attention Awareness Scale* (*a* = .82), as in Study 1.

#### Competitive-Jungle Beliefs

Next, participants completed the 10-item *Competitive-Jungle Beliefs Scale* (*CJB*) developed by Duckitt et al. (2002) [39] to measure individual differences in perceptions that the social world is a competitive jungle in which the powerful win over the weak. Examples of items are: “Winning is not the first thing; it’s the only thing” and “You know that most people are out to ‘screw’ you, so you have to get them first when you get the chance.” Ratings were made on 7-point scales, ranging from 1 = *completely disagree* to 7 = *completely agree*. A composite competitive-jungle beliefs score was computed by averaging responses (*α* = .88); high values indicate higher competitive-jungle beliefs.

#### Dangerous-world beliefs

Participants also completed the 10-item *Dangerous-World Beliefs Scale* (*DWB*) developed by Duckitt et al. (2002) [39] to measure individual differences in perceptions that the social world is a dangerous place in which in-groups’ norms and values are seriously threatened. Examples of items are: “There are many dangerous people in our society who will attack someone out of pure meanness, for no reason at all” and “Every day society becomes more lawless and bestial, and a person’s chances of being robbed, assaulted, and even murdered go up and up.” Ratings were made on 7-point scales, ranging from 1 = *completely disagree* to 7 = *completely agree*. A composite dangerous-world beliefs score was computed by averaging responses (*α* = .79); high values indicate higher dangerous-world beliefs.

#### Social dominance orientation

Subsequently, we asked participants to complete the 8-item short version of the *SDO Scale* (*a* = .90) used in Study 1.

#### Right-wing authoritarianism

Furthermore, we asked participants to complete the 10-item *RWA Scale* [42], which assesses individual differences in RWA. Examples of items are: “What our country really needs is a strong determined leader who will crush evil and take us back to our true path” and “The facts on crime, sexual immorality, and the recent public disorders all show we have to crack down harder on deviant groups and troublemakers, if we are going to save our moral standards and preserve law and order.” Ratings were made on 7-point scales, ranging from 1 = *completely disagree* to 7 = *completely agree*. A composite RWA score was computed by averaging responses (*α* = .85); high values indicate higher RWA.

#### Support for tax progressivity

Finally, we measured participants’ support for a progressive tax system through the 5-item *Preference for a Progressive Tax System Scale* (*TP*; [47]). Examples of items are: “The only fair way is to collect more tax from rich people” and “Tax rates ought to be increased moving from the low-income group to the high one.” Ratings were made on 7-point scales, ranging from 1 = *completely disagree* to 7 = *completely agree*. A composite support for tax progressivity score was computed by averaging responses (*α* = .84); high values indicate stronger support for a progressive tax system.

### Results

Correlations among measures are reported in Table 2.

**Table 2:** Intercorrelations, means, and standard deviations for scores on Mindfulness (MAAS), Competitive-Jungle Beliefs (CJB), Dangerous-World Beliefs (DWB), Social Dominance Orientation (SDO), Right-Wing Authoritarianism (RWA), and support for Tax Progressivity (TP).

	*M*	*SD*	(1)	(2)	(3)	(4)	(5)	(6)
(1) MAAS	4.09	.75	-					
(2) CJB	2.78	1.06	-.24***	-				
(3) DWB	4.16	.94	-.03	.06	-			
(4) SDO	2.26	1.12	-.15***	.62***	-.02	-		
(5) RWA	3.15	1.17	.004	.34***	.31***	.45***	-	
(6) TP	5.04	1.29	.006	-.25***	-.04	-.32***	-.18***	-

*Note*. Study 2 (*N* = 722)

*** *p* < .001.

**Fig 2:**
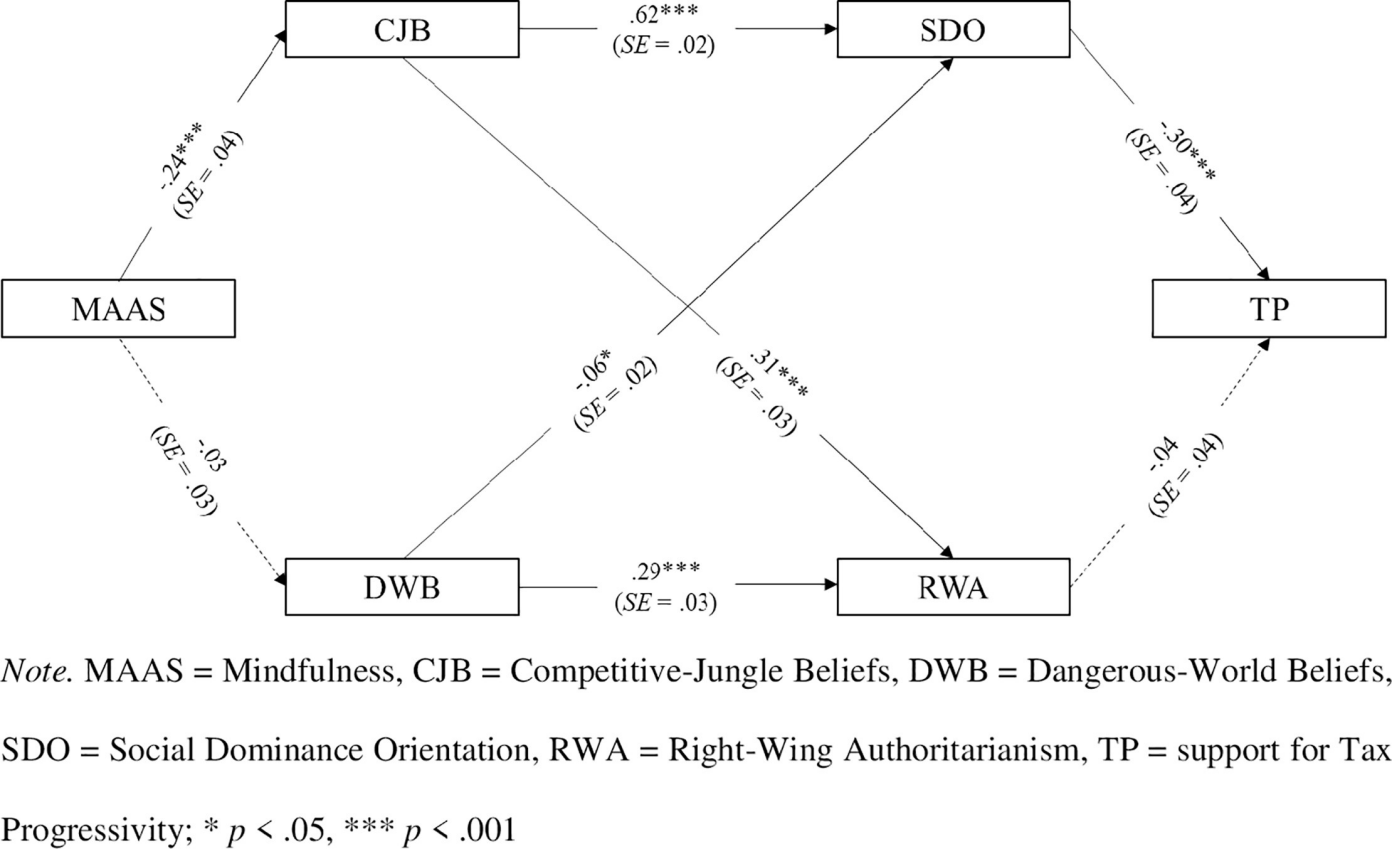
Path analysis model with sequential mediators in Study 2 (*N* = 722): Standardized parameters.

We expected mindfulness to be negatively related with competitive-jungle beliefs and then, SDO. In turn, a lower SDO would be positively associated with support for a progressive tax system (*H2*). We also expected a weaker pathway from mindfulness through dangerous-world beliefs and RWA to relate with support for a progressive tax system (*H2a*). In the model, mindfulness (i.e., the independent variable X) impacted on both competitive-jungle beliefs and dangerous-world beliefs (i.e., the mediators M1), which impacted on the two ideological attitudes, SDO and RWA, respectively (i.e., the mediators M2). Both SDO and RWA, in turn, impacted on support for a progressive tax system (i.e., the dependent variable Y). We tested such a model using Lavaan [48], an R package for Structural Equation Modelling, by means of the RStudio graphical interface [49]. Results of the path analysis model with sequential mediators are summarized in Fig 2.

The model showed good fit: *χ^2^* (5) = 14.744, *p* = .01, with fit indices that met the desired benchmarks: RMSEA = .055, *95% CI* [.024, .089]; CFI = .99. Mindfulness related significantly, and negatively, with competitive-jungle beliefs, *β* = -.24, *SE* = .04, *z* = -6.79, *p* < .001, *95% CI* [-.3115, -.1719], meaning that mindful people were less likely to believe that the social world is a competitive place in which only those who have power can “survive” and win. Instead, mindfulness was not significantly related with dangerous-world beliefs, *β* = -.03, *SE* = .03, *z* = -1.08, *p* = .28, *95% CI* [-.1043, .0300]. Note that this latter CI did not overlap with the one for the mindfulness–competitive jungle link. Consistent with the dual-process model’s predictions [39], competitive-jungle beliefs related significantly, and positively, with SDO, *β* = .62, *SE* = .02, *z* = 22.52, *p* < .001, *95% CI* [.5724, .6815]. As is often found, a smaller positive relationship was found between competitive-jungle beliefs and RWA, *β* = .31, *SE* = .03, *z* = 9.19, *p* < .001, *95% CI* [.2514, .3875], showing that people who endorsed competitive-jungle beliefs were also likely to be higher in RWA. Dangerous-world beliefs related significantly, and positively, with RWA, *β* = .29, *SE* = .03, *z* = 9.34, *p* < .001, *95% CI* [.2294, .3511]. A much smaller and *negative* relationship was found between dangerous-world beliefs and SDO, *β* = -.06, *SE* = .02, *z* = -2.11, *p* = .03, *95% CI* [-.1158, -.0044], showing that people who possessed dangerous-world beliefs were less likely to endorse SDO—attesting to the different processes that make RWA and SDO distinct, although related. SDO related negatively with support for progressive taxation, *β* = -.30, *SE* = .04, *z* = -7.39, *p* < .001, *95% CI* [-.3819, -.2219]; that is, people with greater SDO were less supportive of a progressive tax system. Instead, RWA was practically unrelated with support for a progressive tax system, *β* = -.04, *SE* = .04, *z* = -1.04, *p* = .29, *95% CI* [-.1215, .0367]. It should be noticed that the CI for this latter coefficient was well outside the CI for the regression of support for a progressive tax system on SDO, lending support to *H2a*.

Importantly, the positive indirect relation between mindfulness and support for a progressive tax system through competitive-jungle beliefs and then, SDO, was significant, although small, *β* = .04, *SE* = .009, *z* = 4.93, *p* < .001, *95% CI* [.0275, .0639]. Conversely, mindfulness did not significantly relate with support for a progressive taxation through dangerous-world beliefs and then, RWA, *β* = .0004, *SE* = .0006, *z* = 0.74, *p* = .45, *95% CI* [-.0007, .0016]. As predicted by *H2*, Study 2 suggested thus that the positive relation between mindfulness and support for a progressive tax system is primarily attributable to the motivational path of SDO, which follows competitive-jungle beliefs. This competitive–dominance motivational path, compared to the path pertaining to a threat–control motivation (i.e., DWB and RWA), was found to be more strongly associated with support for progressive taxation, *β*_*diff*_ = .04, *SE* = .009, *z* = 4.79, *p* < .001, *95% CI* [.0270, .0640]. This is consistent with the notion that the relation between mindfulness and support for tax progressivity was channeled by competitive-jungle beliefs and then, SDO, but significantly less by dangerous-world beliefs and then, RWA, as anticipated in *H2a*.

## General discussion

Results presented in this article consistently demonstrated the direct and indirect relations between mindfulness, (a) tax evasion intentions, and (b) support for tax progressivity. These results add to the mindfulness literature by providing new insights on the motivational paths for how mindfulness may relate to citizens’ attitudes and behavioral intentions toward taxes and the tax system. Although studies on citizens’ tax attitudes and behaviors are abundant [50–54], to the best of our knowledge this is the first attempt to investigate whether and how mindfulness relates with people’s tax-related preferences and behavioral intentions.

In Study 1, we found that mindfulness negatively relates with tax evasion intentions through lower SDO. This is consistent with previous results [22] showing that SDO mediates the positive impact of mindfulness on pro-environmental attitudes and behaviors.

In Study 2, we dug deeper on mechanisms, and found that the relation between mindfulness and support for progressive taxation is channeled by competitive-jungle beliefs and, in turn, SDO; instead, there is no mediation through the pathway running through dangerous-world beliefs and RWA. By finding that the relation between mindfulness and support for progressive taxation is primarily attributable to the motivational path of SDO, these results dovetailed with previous findings [22] and provided an extension of Study 1. It appears that mindfulness is more associated with the SDO path than with the RWA path. This is because of the emphasis of mindfulness on pro-sociality and its associated beneficial effects [12], which we see as more consistent with the SDO focus on hierarchical intergroup relations. Instead, the importance of conformity and traditional normative values—along with intragroup hierarchy—makes the pathway running through RWA comparatively less apt to relate with tax attitudes and intentions. These results contribute thus to the body of knowledge about how mindfulness is more likely to produce pro-social effects [12], and they may stimulate future research in this vein.

Interestingly, we found a significant negative association between mindfulness and SDO, which stems from competitive-jungle beliefs, whereas mindfulness is unrelated with RWA, which stems from dangerous-world beliefs. One possible explanation for these results could start by remembering how Edward Burke conservatism viewed history and time: “History is a pact between the dead, the living, and the yet unborn.” RWA and its concern with tradition and time-hallowed practices may lead individuals to conceptualize time as a seamless dimension: time is mentally represented as a linear path in which past, present, and future are not sharply separated from one another, but rather merged all together into a continuous time flow. This conceptualization of time may fit well with the disposition of mindfulness to “stay in touch” with whatever individuals are experiencing moment by moment—hence, a positive link may exist between conservatives’ conceptualization of time and mindfulness. However, RWA is concerned with the dangers a changing world poses to time-hallowed traditions, and such a risk-averse and prevention focus pulls away from sustained attention to the present moment in favor of preoccupied focus on the future. These features of conservatism and RWA should relate negatively with mindfulness. Hence, we may conjecture that the observed lack of association is the end result of opposites cancelling each other—associations between RWA and dangerous-world beliefs on the one side, and with mindfulness on the other.

Although further investigations are needed, these two studies put mindfulness on the agenda of taxation research as an important issue to consider. Moreover, they shed light on motivational pathways through which being mindful relates with both tax evasion intentions and support for a progressive tax system. Our results represent solid ground for future examinations of socio-psychological factors responsible for citizens’ tax evasion intentions and support for a progressive tax system. A deeper understanding of such factors is not only theoretically relevant—it is indeed key for promoting pro-social attitudes and behaviors of taxpayers, as well as practical interventions aimed at ameliorating economic inequality.

## Limits and future directions

We focused on a single country, i.e., Italy, and recruited participants from the Italian population. Specificity of the country-related context is a double-edged sword, with potentially positive but also limiting consequences. On the positive edge, it enables us to investigate the link between mindfulness, (a) tax evasion intentions, and (b) support for progressive taxation in an environment where potentially relevant factors affecting tax attitudes and behaviors are constant, without the complexities that would be added by considering the variability of tax codes across different national contexts. On the negative edge, results obtained in a single specific context are, of course, limited, and surveying participants who live under different tax systems would have strengthened the research design. Beyond the domain of progressive taxation, it could be also useful for both research and practice to investigate our hypotheses by considering different taxation-related dependent variables such as flat tax rate and consumption taxes.

Moreover, it would be desirable to move toward a causal analysis of the predictive role of being mindful on tax-related outcomes. In this direction, future research could include short mindfulness trainings, self-administered via web applications and smart technologies [55], as experimental manipulation of mindfulness in the research design. This is not a perfunctory call for experimentation, but—in this context—it is relevant in terms of applicability of our results: given that tax evasion and tax progressivity entail important economic and social consequences, investigating short mindfulness trainings as potential tools for promoting pro-sociality among taxpayers is a major economic and political issue.
